# In-Depth Bicycle Collision Reconstruction: From a Crash Helmet to Brain Injury Evaluation

**DOI:** 10.3390/bioengineering10030317

**Published:** 2023-03-02

**Authors:** Xiancheng Yu, Claire E. Baker, Mike Brown, Mazdak Ghajari

**Affiliations:** 1 HEAD Lab, Dyson School of Design Engineering, Imperial College London, London SW7 2AZ, UK; 2 Advanced Simtech Limited, Stratford upon Avon CV37 8NF, UK

**Keywords:** biomechanics, traumatic brain injury, road traffic accident, accident reconstruction, simulation, impact test

## Abstract

Traumatic brain injury (TBI) is a prevalent injury among cyclists experiencing head collisions. In legal cases, reliable brain injury evaluation can be difficult and controversial as mild injuries cannot be diagnosed with conventional brain imaging methods. In such cases, accident reconstruction may be used to predict the risk of TBI. However, lack of collision details can render accident reconstruction nearly impossible. Here, we introduce a reconstruction method to evaluate the brain injury in a bicycle–vehicle collision using the crash helmet alone. Following a thorough inspection of the cyclist’s helmet, we identified a severe impact, a moderate impact and several scrapes, which helped us to determine the impact conditions. We used our helmet test rig and intact helmets identical to the cyclist’s helmet to replicate the damage seen on the cyclist’s helmet involved in the real-world collision. We performed both linear and oblique impacts, measured the translational and rotational kinematics of the head and predicted the strain and the strain rate across the brain using a computational head model. Our results proved the hypothesis that the cyclist sustained a severe impact followed by a moderate impact on the road surface. The estimated head accelerations and velocity (167 g, 40.7 rad/s and 13.2 krad/s^2^) and the brain strain and strain rate (0.541 and 415/s) confirmed that the severe impact was large enough to produce mild to moderate TBI. The method introduced in this study can guide future accident reconstructions, allowing for the evaluation of TBI using the crash helmet only.

## 1. Introduction

Cycling is among the most popular modes of active mobility. Particularly, during the COVID-19 pandemic, there has been a significant increase in cycling. In England from 2019 to 2020, there has been a 26% increase in average cycling trips and a 63% increase in total miles cycled [1]. Despite the many health benefits associated with cycling, cyclists are at high risk of injuries in road traffic collisions (RTCs). The 2020 annual report of road casualties in Great Britain shows that 141 pedal cyclists were killed, 4215 were seriously injured and 11,938 slightly injured in 2020 across Great Britain [2]. The number of cyclist fatalities in 2020 rose by 41% from 2019.

Traumatic brain injury (TBI) is the leading cause of death and admissions to hospital among bicycle-related injuries [3]. In bicycle collisions, the head can impact with another vehicle, road-side infrastructure or the road surface, which may lead to skull fractures, intracranial bleeding and diffuse axonal injuries (DAI). A recent study has analysed the UK’s Road Accident In-Depth Studies (RAIDS) database, which documents a subset of road traffic collisions in the UK, for the prevalence of TBI across different road users [4]. This study has shown that of the 112 cyclists involved in collisions between 2013 and 2020, 14.3% sustained a skull fracture, 7.1% sustained a subdural haematoma (SDH), 23.2% sustained a subarachnoid haemorrhage (SAH) and 11.6% sustained a focal brain injury [4]. The rate of DAI was not reported as the clinical data in RAIDS are based mainly on computed tomography (CT) imaging, which often misses diffuse axonal injuries [4,5]. Surprisingly, 51% of the cyclists did not wear a helmet at the time of the collision, and these non-helmeted cyclists had a much higher risk of skull fracture and SDH, as supported by other studies [6].

Clinical diagnostic tools (e.g., CT/MRI scans) and score-based injury assessment (e.g., the Mayo classification system for TBI severity and the Glasgow coma scale) are frequently used to assess the severity of TBI. However, these clinical diagnostic tools do not capture all TBI cases or presentations. The detection is mostly accurate for acute brain injury pathologies, for example haematoma and focal brain injury; however, these methods often miss diffuse and microscopic injuries, such as DAI [7,8], or they have poor correlation with long-term outcome, such as cognitive impairment [9]. In addition, TBI can trigger neurodegenerative diseases such as chronic traumatic encephalopathy (CTE) [10,11,12]. These diseases can develop years or even decades after single or repetitive head impacts [13,14]. These TBI diagnostic challenges have created much controversy in brain injury legal cases when there is no clinical evidence supporting brain injury. While self-reported brain injury symptoms by patients can be questionable and easily challenged, there is an urgent need to develop more reliable and convincing methods to assess brain injuries.

One approach to evaluate brain injury is to reconstruct the head impact event, such as in road traffic collisions (RTCs), and determine the level of mechanical exposure measured through injury risk metrics based on head kinematics. Such reconstructions are usually conducted with multi-body or full body finite element simulations [15,16,17,18,19], whose initial conditions are defined based on an accident report or video footage [18]. These studies have provided invaluable information to enhance our understanding of the human body dynamics and head impact kinematics during a collision. However, their prediction of head impact conditions may not be accurate for two main reasons. First, the majority of RTCs do not have video footage or their footage is low-resolution and consequently cannot be used to validate the model prediction of body kinematics. Secondly, while a small number of studies compared the model prediction against available evidence including video footage, scene photographs, CCTV or dashcam footage, the aim of these simulations was to match the overall human body motion with the video footage rather than the precise head impact conditions, which often are unclear in video footage.

In most helmeted cyclist collisions, the crash helmets are preserved. These helmets contain key signatures of the head impacts and can be very useful for experimental reconstruction. A few previous studies have reconstructed motorcycle or bicycle collisions by impacting the identical and undamaged helmets to match the damage of the crash helmet [20,21,22,23]. For example, Bland et al. [21] have quantified the helmet damage by CT scans and conducted oblique impact tests to reconstruct 18 damaged helmets from bicycle collisions. They found that the majority of impacts were oblique, producing large rotational acceleration and velocity of the head. Such oblique impact tests have not been used in earlier bicycle helmet reconstructions [22,23]. These studies have focused on the headform kinematics results of the reconstructed tests, which provided invaluable information about brain injury risk. However, there is little information about how to choose and iterate through impact conditions, i.e., impact speed, angle and location, based on helmet damage quantification. In addition, no photographic comparison has been presented in previous studies to confirm the degree of similarity between reconstruction and crash helmets from real-world incidents in terms of the damage patterns.

In this study, we experimentally reconstructed a cyclist’s head impact based on the crash helmet, which was the only physical evidence from the collision. The cyclist reported cognitive impairment after head impact, which was not detected in clinical examinations. The first aim was to test if the impact conditions can be estimated from the analysis of the helmet damage patterns, including the crushed area/depth, cracks, pits and scrapes. The second aim was to test if the damage pattern can be replicated on identical helmets by conducting a small number of normal and oblique impacts on the helmets guided by the estimated impact conditions. The final aim was to determine the likelihood of traumatic brain injury based on the headform kinematics measures from the impact tests and the brain deformation from computational modelling.

## 2. Methods

### 2.1. Background of the Accident

A bicycle helmet damaged in a road traffic collision was provided to Imperial College HEAD lab by Advanced Simtech, an accident reconstruction specialist who were instructed by the injured rider’s legal team. According to the brief police report on this case, the vehicle’s driver failed to see the cyclist when approaching a roundabout. As such, the car pulled out directly into the path of the cyclist. This resulted in a collision, whereby the cycle struck the vehicle’s front offside wing, resulting in the cyclist being projected over the frontal vehicle structure, causing the cyclist to land on the road surface in a position beyond the car. The cyclist states that they would have been travelling between 6.7–8 m/s (24–29 km/h) when the collision occurred. The cyclist’s injuries were initially recorded as headache, lower back and shoulder pain, fractures of the L4 vertebral body of the back, soft tissue injuries to the left knee and shoulder, and some memory loss. After the accident, the cyclist additionally began to report cognitive deficits.

The accident and emergency records report no loss of consciousness at the scene. However, the cyclist has no recollection of the incident other than a patchy recollection of events once they were admitted to the hospital. The driver’s insurance company questioned the level of force involved in this collision, suggesting that the speeds would have been much lower than those provided by the cyclist. As such, the cyclist’s legal team were looking for a way to provide additional engineering analysis that could support their client’s case. 

### 2.2. Analysis of the Helmet Damage

**Figure 1:**
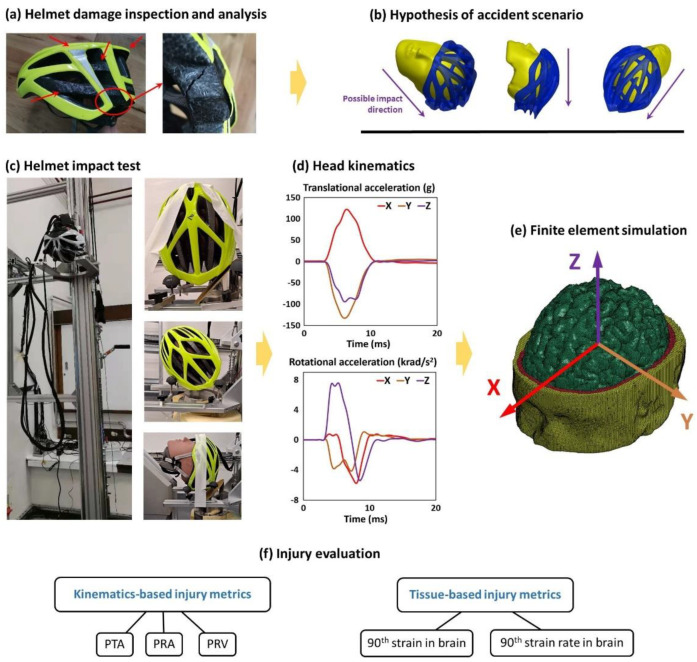
(**a**) The cyclist’s helmet was firstly inspected and all damages were analysed. (**b**) We then hypothesised the accident scenario including the head trajectory and range of head impact speed and direction. (**c**) Next, we placed the helmeted headform onto a U-shaped platform, which was raised to a certain height to generate a desired impact speed from free fall. The headform’s orientation and the impact speed were chosen based on the hypothesised accident scenarios. (**d**) For each test, three translational and three rotational acceleration time-history data were recorded with the HIII headform. (**e**) These acceleration data were then applied to a detailed finite element model of human head to determine the brain strain and strain rate. (**f**) Finally, we evaluate the brain injury using two types of injury metrics: kinematics-based injury metrics and tissue-based injury metrics.

We inspected the cyclist’s helmet for all damage types and locations (Figure 1a). As the helmet did not have obvious wear and tear marks outside the impacted area, we assumed that the helmet did not have previous damage before the accident, which was also confirmed by the cyclist. We divided the damage into four types: helmet liner crushing, liner cracks, surface scrapes and small localised damage. From this analysis, we formed hypothesises about the accident scenario, head trajectory and range of head impact speed and direction (Figure 1b).

### 2.3. Helmet Impact Testing

We used the Imperial College HEAD Lab’s helmet test rig (Figure 1c) to test the hypothesised head impact conditions and reconstruct the damage profile seen on the cyclist’s helmet. The helmeted headform was placed onto a U-shape platform and raised to a certain height to generate a desired impact speed from free fall (Figure 1c). The impact anvil and the headform’s orientation were changed to accommodate different testing scenarios (Figure 1c). The cyclist reported head impact to the road surface, not the vehicle body. This claim was supported by the small pits damage of the helmet, which were created by the protruding road aggregates. Therefore, we used steel anvils to model the rigidity of the road surface. Here, we used both the flat anvil and 45° oblique anvil to conduct normal and oblique impact tests. The oblique anvil was covered with a 80 grit sandpaper to simulate the road asphalt surface [24]. 

We used a 50th percentile Hybrid III headform for the experiments (Figure 1c). The headform was instrumented with an array of nine miniature piezoelectric accelerometers, mounted inside the headform in the 3-2-2-2 arrangement. This allowed us to measure both linear and rotational accelerations at the centre of mass of the headform. Figure 1d shows the linear and rotational accelerations time-histories measured in Test 8. The acceleration data were recorded at a 50 kHz frequency and filtered with a 4th order Butterworth low-pass filter with a 1 kHz cut-off frequency. We used masking tape to fix the helmeted headform onto the platform, maintaining the helmet’s position and orientation during the free fall. The masking tape was pre-cut at several points to ensure it could tear easily during the impact. We have previously verified that the tape fixture has minimal effect on the headform motion [24].

We conducted 10 tests to recreate the damage produced by the severe impact. After each test, the helmet sample was inspected and its damage was compared with the cyclist’s helmet, helping us to improve the impact conditions for the next test. All the helmets were tested once, ensuring damage was not accumulated.

### 2.4. Finite Element Modelling

In order to predict brain deformation during the collision, we used a 3D finite element (FE) model of the human head, which was previously developed at the Imperial College HEAD lab (Figure 1e) [25,26]. The head FE model was developed from the MRI scans of a healthy 34-year-old male. Therefore, the model represents key tissues at a high resolution, such as scalp, skull, brain, cerebrospinal fluid, and falx. Particularly, the anatomical features of the grey and white matter, such as sulci and gyri, are represented in detail, ensuring that the effects of these anatomical details are considered in the simulations. The material models and parameters of the various tissues are explained in detail in our previous study [18,25,27]. Using this head model, we simulated two tests that best replicated the damage on cyclist’s helmet. Here, we assumed that the skull is rigid due to its small deformation in helmeted impacts. The three translational and three rotational accelerations measured in helmet experiments were applied to the skull at the centre of gravity of the head. The first 30 ms after initial contact was simulated, which was enough for the brain to experience the peak deformation. The simulations were conducted using the nonlinear hydro-code LS-DYNA R11 [28]. The simulation results were postprocessed to obtain the strain and the strain rate across the brain [25,29]. Strain is a measure of how much the brain element has been deformed with respect to its shape in the undeformed configuration. For each element of the model, we determined the maximum principal value of the Green–Lagrange strain tensor during the simulation [2]. Next, we determined the maximum principal value of the total time derivative of the Green–Lagrange strain tensor, which is the strain rate. The strain rate represents how fast the brain element deforms.

### 2.5. Brain Injury Metrics and Evaluation

We used three kinematics-based injury metrics and two tissue-based injury metrics to evaluate brain injury (Figure 1f). Skull fracture, subdural haematoma and diffuse injuries are commonly seen pathologies after head collisions. These injuries can be evaluated by kinematic-based head injury metrics. Here, we used three kinematic-based head injury metrics: the peak translational acceleration (PTA), the peak rotational acceleration (PRA) and the peak rotational velocity (PRV). The PTA is used in all helmet testing standards, and it can predict the risk of skull fracture/focal injury [30,31]. The PRA is adopted for predicting the risk of subdural haematoma (SDH) [32,33]. The PRV is adopted for predicting diffuse injuries, such as diffuse axonal injury (DAI) [34]. The two tissue-based injury metrics were the 90th percentile values of the maximum strain and strain rate of the entire brain, which are commonly used to predict diffuse axonal injuries [24,35,36,37].

## 3. Results

### 3.1. Helmet Damage Profile and Hypothesised Accident Scenario

All observed damages are listed in Table 1. We hypothesised that the damage was produced by a severe impact, a moderate impact and several mild impacts, which are detailed below.

#### 3.1.1. The Severe Impact

**Figure 2:**
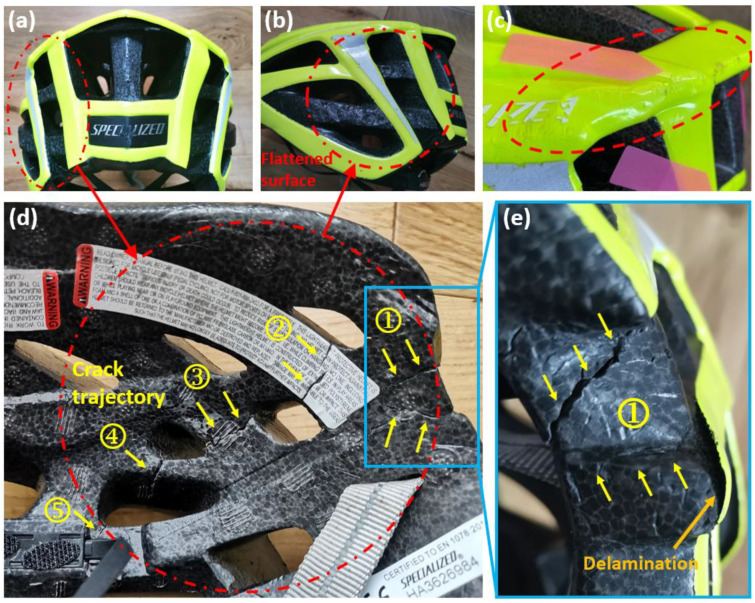
(**a**,**b**) The flattened surface, (**c**) the scrapes and buckling shell and (**d**,**e**) crack trajectory of the cyclist’s helmet (the numbers mark the cracked regions).

We observed a flattened region with a diameter of 75 mm on the rear left side of the helmet (Figure 2a,b). We determined a 4.9 mm crush depth by comparing the flattened region with the intact region on the rear right of the helmet. In addition, part of the helmet shell delaminated from the liner (Figure 2e). 

The severe impact also cracked the liner, visible on the interior of the liner. Figure 2d shows the crack trajectory, consisting of four major cracked regions. There are 5 cracks, including two in region 1 and one in each of the other regions (Figure 2e). These cracks initiated from the inner surface and propagated to the outer surface. The opening of cracks in regions 1–4 is large (>0.5 mm). Crack regions 1–4 are within the area of the outside flattened surface. The opening of the crack in region 5 is small. 

On the outside upper edge, the impact created scrapes and caused shell buckling (Figure 2c). The scrapes suggest that the cyclist’s head had tangential velocity to the road, in addition to the normal velocity that created the flattened surface. 

From this analysis, we hypothesised that this area sustained the initial helmet–road impact and absorbed the majority of the impact energy. We also hypothesised that the internal cracks in the liner were produced by bending of the liner produced by the head compressing against the liner during the impact on the hard surface of the road. In addition, the scrapes and shell buckling indicated that the impacts were oblique rather than normal.

#### 3.1.2. The Moderate Impact

**Figure 3:**
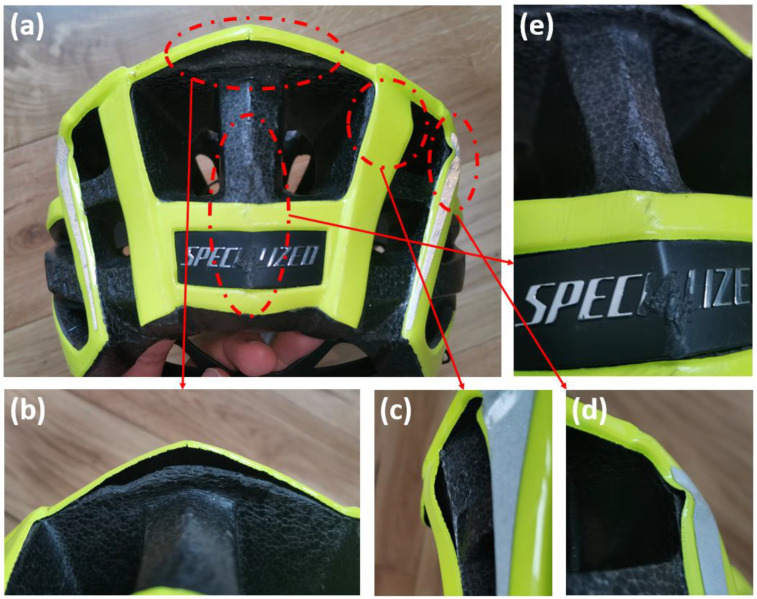
(**a**) Damages produced by the moderate impact and minor impacts, (**b**) ESP crush at the rear top region, (**c**,**d**) shell delamination and buckling and (**e**) pits and scratches on the rear region.

We observed foam crushing at the rear top of the helmet, likely caused by a moderate secondary impact. As shown in Figure 3a,b, the rear top region was crushed and delaminated from the shell. After the impact, the shell rebounded to its original position and shape. We determined a 6 mm crush depth by measuring the distance between the liner and shell. Unlike the flattened surface, this crushing was localised to the rear edge of the liner thus having much smaller volume, suggesting a low energy impact at this point. Hence, we hypothesised that after the severe impact at the flattened area, the cyclist’s head experienced another impact but at lower energy.

#### 3.1.3. Mild Impacts

We observed several localised areas of minor damage on the helmet (Table 1). More specifically, there were two buckled regions in the shell at the rear right region of the helmet (Figure 3c,d), which are likely to have been produced by a tangential force due to sliding of the helmet on the road. On the rear side of the helmet, the EPS had some small pits and shell showed scratches (Figure 3e). This damage is mild, hence it should have been produced by low-energy, minor impacts. Hence, we hypothesised that after the severe and moderate impacts, the helmet had a few more impacts on the road but at considerably lower energies.

### 3.2. Helmet Impact Testing Results

We conducted 10 tests to recreate the damages from the severe impact and 2 tests for the moderate impact.

#### 3.2.1. Reconstruction of the Severe Impact

Table 2 summarises the impact conditions (anvil and speed) and results of 10 tests, which were performed to recreate the damage from the severe impact. We report the crush depth of the liner, whether the cracks seen on cyclist’s helmet were recreated, and whether the impact produced any additional cracks.

We first conducted three impacts using the flat anvil, with impact speeds ranging from 5.8 to 6.9 m/s. This was to recreate the crushing distance seen in the crash helmet. However, the created crush depth was larger than the cyclist’s helmet and the crack trajectories were different. Guided by these results, from Test 4 onwards, we used the oblique anvil and used impact velocity with a lower normal component. In these tests, we varied both the helmet orientation and the impact speed. 

The impact speed in Test 4 was 6.4 m/s, but it produced significantly less damage compared with the cyclist’s helmet, with only two cracks recreated. In addition, the crush depth (2.7 mm) was much smaller than observed on the cyclist’s helmet (4.9 mm). This indicates that the head impact velocity had to be larger than 6.4 m/s. Therefore, we increased the impact speed from Test 5 onwards.

**Figure 4:**
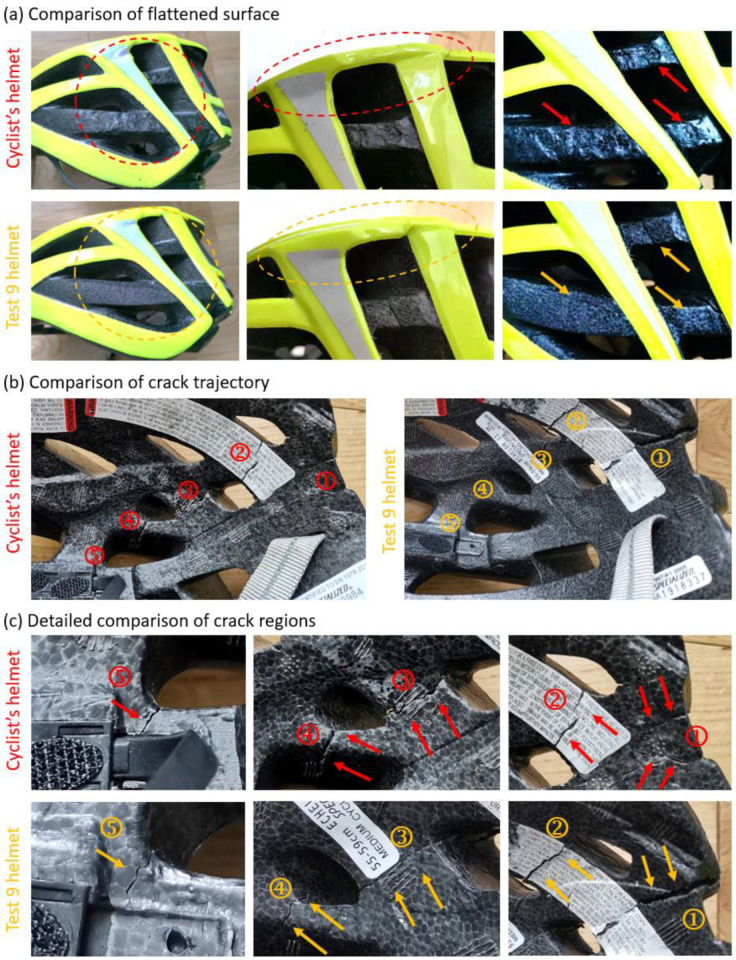
Comparison of cracks between the cyclist’s helmet and Test 9 (the numbers mark the cracked regions).

Among all the tests, the helmet damage in Test 9 was the closest to the cyclist’s helmet. Figure 4a compares the flattened surface of cyclist’s helmet and the helmet in Test 9, showing excellent agreement. The crushed area of the helmet matched well with the cyclist’s helmet, except that the crushed surface was flat with no pits in contrast to the cyclist’s helmet. This is likely due to the smooth surface of the oblique anvil in contrast to the road surface. The crush depth in Test 9 was 5.2 mm, which was slightly higher (6.1%) than that observed on the cyclist’s helmet (4.9 mm). Test 9 also recreated scrapes and inward bending of the shell (Figure 4a), supporting our hypothesis that the severe impact was oblique. Overall, the damage created in Test 9 was very similar to the damage from the accident.

Test 9 also recreated all the five crack regions observed inside the liner (Figure 4b). Most of the recreated cracks matched the cyclist’s helmet; the crack in region 2 is slightly shifted from that of the cyclist’s helmet. The detailed comparisons of each crack region are shown in Figure 4c. In Test 9, the location and size of cracks in regions 1, 3, 4 and 5 agree well with the cyclist’s helmet. Noticeably, the crack in region 2 is of a similar size to the crack on cyclist’s helmet, which suggests an accurate estimation of the impact energy.

Test 8 and 10 used the same impact speed as Test 9, but different head orientations. This change led to creation of additional cracks on the outer surface of the foam. In addition, in the inner surface, the crack openings were larger than those in the cyclist’s helmet. These suggest that the damages in Test 8 and 10 are more severe than in cyclist’s helmet.

#### 3.2.2. Reconstruction of the Moderate Impact

**Figure 5:**
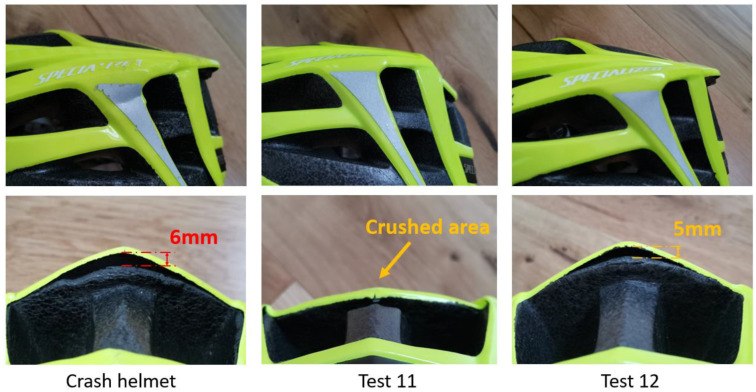
Comparison between cyclist’s helmet and Tests 11 and 12.

Tests 11 and 12 were performed to recreate the damage at the rear site. The test conditions and results are summarised in Table 3. Figure 5 shows the comparison between cyclist’s helmet and the helmets from Test 11 and 12. The results show that the 8 m/s impact speed in Test 11 created severe damage at the rear site, crushing a large area and creating several localised cracks. This damage is much more severe than that observed on the cyclist’s helmet. The impact speed of 3.2 m/s (corresponding to a fall from 0.5 m) in Test 12 produced similar crush damage to cyclist’s helmet. In addition, similar to the cyclist’s helmet, after this impact the shell separated from the liner and rebound to its original shape. The crushing depth (distance between the shell and crushed foam) was 5 mm in the test, which is slightly smaller than that in cyclist’s helmet (6 mm). These results confirm a lower energy oblique impact at the rear site. 

### 3.3. Brain Injury Metrics and Injury Evaluation

**Figure 6:**
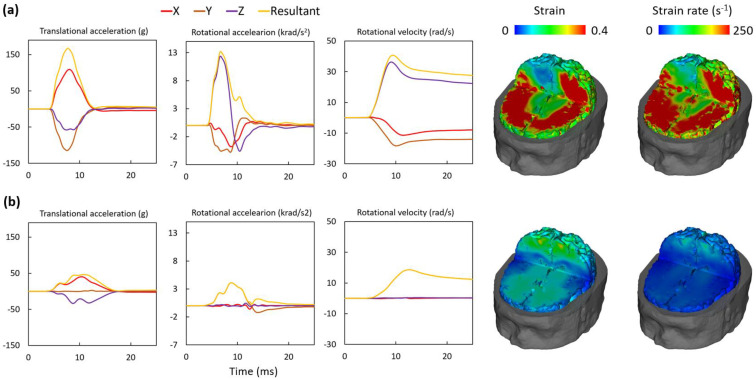
Headform kinematics and brain deformation contours for (**a**) Test 9 and (**b**) Test 12 (note that the rotational velocity curve about Y axis overlaps with the resultant curve).

We recreated the severe damage in Test 9 and moderate damage in Test 12. Therefore, we report the headform kinematics measured in both of these tests. Figure 6 shows the translational and rotational accelerations, the rotational velocity and the brain strain/strain rate contours predicted by the brain model. Notably, our results show that the impact duration was approximately 10 ms, which is expected in helmeted head impacts against rigid surfaces [38]. Overall, Test 9 produced much higher values in the headform kinematics and brain deformation than Test 12. For example, a large volume of the brain undergoes a strain of more than 0.4 and a strain rate of more than 250 s^−1^ in Test 9. Test 12, however, produced a much smaller strain and strain rate across the brain. 

Table 4 lists the values of the three kinematics-based and two tissue-based injury metrics. The PTA was lower than the pass/fail limit prescribed in helmet standards, 250–300 g [39]. This limit, however, is suggested for life-threatening TBI. The limit for mild–moderate TBI is lower. A 106 g value for the peak translational acceleration is suggested to be equivalent to an 80% probability of mild TBI [40]. The PTA in Test 9 is 58% larger than this threshold value. The PTA in Test 12 is only 46 g, which is substantially below the 25% probability of mild TBI (57 g), therefore is less likely to produce brain injury [40,41].

Rotational motion of the head plays a significant role in shearing brain tissue and damaging axons and blood vessels. Previous post mortem human subjects experiments suggested a 10 krad/s^2^ PRA threshold for bridging veins rupture (leading to SDH) when the pulse duration is shorter than 10 ms [32]. In addition, the 80% probability of sustaining mild TBI is estimated to be 7800 rad/s^2^ [40]. The PRA in Test 9 exceeded both values, suggesting the likelihood of mild TBI and possible SDH. Another study investigated head injury severity using the Abbreviated Injury Scale (AIS) score and showed that the PRA for producing TBI at AIS2-3 was estimated to be 8000–18,000 rad/s^2^ [42]. The recorded value of the PRA in Test 9 falls in this range, suggesting TBI with AIS2+ severity. The PRA in Test 12 is 4102 rad/s^2^, lower than these thresholds.

DAI is a determinant of poor cognitive outcome in TBI patients [43]. One study suggested that a PRV of 46.5 rad/s can produce DAI with AIS4+ severity [34]. In Test 9, the PRV was 40.7 rad/s, suggesting a high risk of DAI. The PRV in Test 12 is 18.6 rad/s, suggesting a much lower risk of DAI from this impact. 

Strain and strain rate have also been widely used to predict TBI at different severities [26]. Animal experiments have suggested a 0.21 strain threshold for producing axonal injury [35]. Another study on American football players found a 0.24 strain limit for an 80% probability of mild TBI [40,41]. Our recent study on rats has shown a strain in excess of 0.3 can produce significant axonal damage (thinning of corpus callosum, and reduction in neurofilaments) [44]. The brain strain contour (Figure 6a) in Test 9 shows that a large volume of the brain experienced strain over 0.4, with the 90th percentile value of the brain strain being 0.54. Previous work has found that the maximum strain rate correlated with the corpus callosum injury and the average value of 10 athletes with concussion is 54 s^−1^ [45]. Another study reconstructed 58 brain injury cases of football players and found the 50% risk of concussion to be a brain strain rate of 48.5 s^−1^ [46]. The strain rate contour and the 90th percentile value of the strain rate (415 s^−1^) in Test 9 largely exceed the values reported in these previous studies. Although there are differences between humans and animals and between different brain models, the combined studies above suggest that the brain strain and strain rate predicted in Test 9 are large enough to produce brain injury.

## 4. Discussion

We successfully reconstructed the cyclist head impact using the crash helmet only. The crash helmet holds signatures of impacts. We mapped its damage profile, which allowed us to hypothesise that the cyclist had sustained a severe and a moderate head impact. By iteratively changing test conditions, i.e., impact speed and angle, we produced damage on the surrogate helmets that closely matched those on the cyclist’s crash helmet. By comparing the values of head kinematic measures, including peak translational and rotational accelerations and the peak rotational velocity, with studies on brain injury thresholds, we estimated that the cyclist was highly likely to have sustained a mild–moderate TBI. This conclusion corroborates with the cognitive deficits reported by the patient, and it led to the patient receiving compensation for the injuries caused by the collision.

Our experimental reconstruction results support the cyclist’s claim of brain injury. Current TBI diagnostic methods can detect large changes in the brain, such as haematoma and possibly DAI, but they may not be sensitive enough to determine milder forms of TBI. Advanced neuroimaging techniques can be used to detect subtle changes in the brain, such as susceptibility weighted imaging and diffusion tensor imaging [25,43,47]. However, it is unclear whether more advanced diagnostic approaches were used for the cyclist in this case study. The cyclist’s clinical report did not support the compensation claim in the legal case, but the cyclist reported cognitive deficits. Based on our reconstruction, we showed that the severe impact should have produced head kinematics and brain strain/strain rate values which were high enough to produce brain damage. This supports the cyclist’s self-report of cognitive deficits. These results helped the cyclist to receive a pre-court settlement by the insurance company with a significantly higher amount of compensation than originally claimed. Although we have reported one case, the reconstruction method and injury metrics used in this study can guide future accident reconstructions, allowing for an evaluation of TBI using the crash helmet alone. 

CT scans are the most common clinical diagnostic approach when a patient is admitted to hospital with a suspected head injury, with MRI sometimes used after the acute phase. However, injury evidence on the imaging can change over time. Some acute injuries may only be detectable within weeks or months after head impact using CT. For instance, a small subarachnoid haemorrhage may be present on a CT scan at the time of head impact but disappears weeks or months later if blood reabsorption occurs. In contrast, some pathologies can only be detected several years after the head impact, such as neurodegenerative diseases (e.g., CTE). Currently, the Glasgow Coma Scale (GCS) score is commonly used to classify TBI severity, with GCS < 13 signifying moderate-severe TBI [48]. Many patients presenting with a GCS in a higher range (13–15) would not necessarily be given a CT/MRI scan in hospital at the time of the incident. As patients can have scans at different times following head impact, it brings much controversy into their legal cases as to what injuries were sustained at the time of head impact. The experimental reconstruction technique we have presented enables insight into the moment of the collision to be gained in the absence of traditional scans, providing a novel diagnostic and legal tool. 

Our tests show that impact speed and angle and head orientation significantly affect the helmet damage. For instance, although Tests 8, 9 and 10 had the same impact speed and angle, the different head orientation produced very different helmet damage. The initial analysis of the helmet damage allowed us to estimate the damage mechanism and head orientation, helping us to use realistic head impact conditions, such as speed and angle. As such, we were able to recreate the damage from the severe and moderate impacts with a limited number of helmets (12 samples). It is likely that in other cases, more impacts will have to be performed to achieve an acceptable reconstruction. Computational modelling is a promising alternative for future reconstructions, allowing us to explore a large range of impact parameters and minimise the number of physical tests required.

In real-world bicycle collisions, most head impacts are oblique [42,49,50]. However, quantifying the impact angle in real-world collisions is very difficult. Previous work has used multi-body simulations to estimate the head impact characteristics in bicycle falls to the ground [51]. Their results show that at a travelling speed of 5.5 m/s, the head impact angle is 36° ± 8° but it increases to 58° ± 6° if travelling speed is increased to 11 m/s. In the present case, cyclist’s travelling speed was reported to be 6.7–8 m/s (24–29 km/h), with the 5.5 to 11 m/s range. Hence, our assumption of the cyclist’s head impact angle of 45° is in agreement with the findings of the multi-body simulations. Moreover, by using the 45° anvil, we produced damage on the helmet that closely matched the cyclist’s helmet, including the wrinkles on the shell. This confirms that the adopted impact angle is appropriate.

Our study has several limitations. First, we quantified the crush damage of the helmet by measuring the crush depth and area. Although these two parameters were averaged from multiple measurements, it may still bring in errors due to the inaccuracy of human visual judgement. In addition, these two parameters represented an approximate evaluation of the absorption of linear kinetic energy while the crush volume is a more accurate and comprehensive measure of energy absorption. Previous studies have used CT scans to quantify not only crush depth and area but also crush volume [21,52,53]. Future work should use such techniques to improve the quantification of the damage. Secondly, we covered the oblique anvil with 80-grit abrasive paper to simulate the road surface, as suggested in previous studies and current helmet testing standards/rating schemes [21,24,54,55]. The flat anvil produced a smooth crush surface on the helmet. This was different to the crash helmet, whose surface has small pits as a result of compression by the protruding road aggregates. It is likely that these protruding road aggregates may restrict the sliding between the helmet and the impact surface, and therefore increase the headform’s rotational motion. This requires further investigation. Thirdly, we used the HIII headform as this headform has been widely used in previous studies [24,38,56,57]. However, it has been shown that its vinyl rubber skin has a larger coefficient of friction against the fabric than human scalp, which may lead to overestimation of head rotational motion [58,59]. Future work should use headforms that have a better coefficient of friction to provide a better estimation of head rotational kinematics [37,60]. Finally, we used an isolated headform, thus ignoring the effects of the neck and the rest of the human body, which has been widely used in previous studies [24,37,38,60,61]. It has been shown that the human body affects the head response, but the effect varies in different impact scenarios [62,63,64,65]. For instance, in head-first impacts, the primary peak loads of the head are less affected by the human body [62,65]. Considering that the impact scenario in the present case is head-first impact and the duration of our tests are short (approximately 10 ms), we did not include a neck or the body in the tests.

In summary, we conducted an in-depth experimental reconstruction of a bicycle head collision using the accident-damaged helmet as the only piece of information. The analysis of helmet damage guided the hypothesised test conditions. This allowed us to reconstruct the helmet damage to a high degree of accuracy within laboratory setting. This work introduces a promising biomechanical approach for evaluating head injury from the crash helmet, providing significant support in brain injury legal cases where clinical evidence is missing. In addition, it enables future head impact reconstruction of cases with limited information about the collision.

## Figures and Tables

**Table 1:** The damages observed on the cyclist’s helmet.

Cause	Damages	Location	Details
Severe impact	Crushed surface	Rear left	1. 75 mm in diameter with small pits, produced by road aggregates compression 2. Crush depth of the flattened surface is 4.9 mm 3. Delamination between shell and liner
	Crack trajectory	Rear left	Five major cracks
	Localised shell buckling	Rear left-top	Localised shell buckling with scrapes
Moderate impact	Crushed rear end	Rear top	1. Crush depth is 6 mm 2. Delamination between shell and the liner
Mild impacts	Delamination	Rear right	1. Delamination between shell and liner 2. No obvious crush of liner
	Localised dents	Rear	Small pits on the EPS form and shell

**Table 2:** Tests for recreating flattened surface/crack trajectory.

Test No	Anvil	Velocity (m/s)	Crush Depth of Flatten Area (mm)	Recreated Cracks in Tested Helmet *					
				1	2	3	4	5	No. of Additional Cracks
1	Flat	5.8	6.2	●	×	●	×	×	2
2	Flat	6.3	6.3	●	×	●	●	×	2
3	Flat	6.9	7	●	×	●	●	●	2
4	Oblique	6.4	2.7	●	×	●	×	×	0
5	Oblique	6.8	6.1	●	×	●	●	●	1
6	Oblique	8	3	●	×	×	×	×	3
7	Oblique	8	3.5	●	●	●	●	×	2
8	Oblique	7.3	5.4	●	●	●	●	●	2
9	Oblique	7.3	5.2	●	●	●	●	●	0
10	Oblique	7.3	5.6	●	●	●	●	●	2

* ● means the crack was recreated while × means not recreated.

**Table 3:** Tests for recreating the crushing at the rear of the helmet.

No	Anvil	Velocity (m/s)	Results
11	Oblique	8	Severe crushing at the rear upper point, which indicates much higher kinematic energy than in accident
12	Oblique	3.2	5 mm crushing, which is close to the 6 mm crushing in the cyclist’s helmet

**Table 4:** Head kinematics summary.

Test No.	PTA (g)	PRA (rad/s^2^)	PRV (rad/s)	90th Percentile Brain Strain	90th Percentile Brain Strain Rate (s^−1^)
9	167	13,206	40.7	0.541	415
12	46	4102	18.6	0.045	178

## Data Availability

Not applicable.
